# Symptom network analysis of depressive and somatic symptoms and suicide risk indicators in treatment-naïve adolescents with first-episode depression

**DOI:** 10.1186/s12888-026-08074-1

**Published:** 2026-04-21

**Authors:** Guixia Liu, Xinning An, Xuebin Wen, Hong Luo, Xinglian Wang, Hao Ren, Xiufen Zhong, Yue Gao, Shixin Yu, Jin Zhang, Ghalia Zainab Jafri, Haitang Qiu

**Affiliations:** 1 Department of Psychiatry, The First Affiliated Hospital of Chongqing Medical University, Key Laboratory of Major Brain Disease and Aging Research (Ministry of Education), Psychiatric Center of Chongqing Medical University the First Affiliated Hospital, Chongqing, China; 2 Department of Sleep and Psychology, Chongqing Health Center for Women and Children, Chongqing, China; 3 Chongqing Changshou District Third People’s Hospital, Chongqing, China; 4 Chongqing Mental Health Center, Chongqing, China

**Keywords:** Depressive disorder, Adolescent, Somatic symptoms, Suicidal ideation, Suicide, Attempted, Network analysis

## Abstract

**Background:**

Depression in adolescents often co-occurs with suicidal ideation and behaviors. In clinical practice, many adolescents with depression report prominent somatic complaints, yet the relevance of these somatic symptoms to different forms of suicide risk has received limited attention.

**Objective:**

This study explored the network among somatic symptoms, depressive symptom dimensions, and three suicide-related indicators—passive suicidal ideation (PSI), active suicidal ideation (ASI), and suicide attempts (SA)—in a sample of treatment-naïve adolescents presenting with first-episode depression.

**Methods:**

This cross-sectional study included 414 adolescents in China. Symptom data were collected using the Hamilton Depression Rating Scale (HAMD-17), Patient Health Questionnaire-15 (PHQ-15), Beck Scale for Suicide Ideation (BSI), and Columbia-Suicide Severity Rating Scale (C-SSRS). A Mixed Graphical Model estimated network structure, node centrality, and predictability for depressive and somatic symptoms and suicide risk indicators.

**Results:**

Cardiopulmonary and pain symptoms occupied central positions within the symptom network. Cardiopulmonary symptoms were weakly connected to passive suicidal ideation, whereas pain symptoms showed weak associations with suicide attempt status.

**Conclusions:**

This cross-sectional symptom network describes how somatic symptom dimensions—particularly cardiopulmonary and pain symptoms—are positioned within a symptom network encompassing depressive symptoms and suicide risk indicators in adolescents with depression. Rather than supporting causal interpretations, these findings highlight the utility of symptom networks as a descriptive framework for organizing somatic symptoms within heterogeneous suicide risk profiles and for informing future longitudinal investigations.

**Clinical trial number:**

Not applicable.

**Supplementary Information:**

The online version contains supplementary material available at 10.1186/s12888-026-08074-1.

## Introduction

Depression in adolescence is often accompanied by a substantial burden of suicide-related illness [1]. Epidemiological studies have shown that around a third of adolescents report having depressive symptoms, but suicide remains one of the leading causes of death among adolescents [2, 3].

The widely accepted “ideation-to-action” framework suggests that suicide occurs via a staged process with ideation and action being subject to only partially overlapping mechanisms [4–6]. However, this framework is largely based on adult samples [7]. During adolescence, cognition, emotion regulation, and behavioral control continue to mature, which may limit the applicability of adult-derived models for characterizing suicide risk in this period [8–12]. Research focusing on treatment-naïve adolescents with depressive illness is particularly scarce.

Much of the existing literature focuses on the cognitive and affective symptoms of depression, and somatic symptoms tend to be overlooked [13–17]. However,nearly half of depressed adolescents clinically report somatic symptoms like fatigue and pain [18–20]. Several studies have reported that a higher burden of somatic symptoms tends to be accompanied by a more severe depressive manifestation and increased suicide risk [21–23]. This sort of phenomenon is more common in the Asian population where they express distress more so in somatic symptoms [24, 25]. Somatic symptoms may be clinically observable features associated with suicide risk, either independently or in conjunction with cognitive and affective symptoms [26].

Although they are clinically meaningful, somatic symptoms are rarely incorporated into current suicide risk literature. There is likely a tendency to conceptualize depression as a unified construct, thereby promoting an apparent homogeneity in symptomatology and obscuring the ways in which suicidal risk may be patterned [27]. Additionally, the contribution of somatic symptom configurations to specific suicide risk indicators is not well delineated. Given the multidimensional nature of somatic and suicidal risk, conventional linear models seldom detect the complex cross-domain associations revealed by network methods [23, 28].

Symptom network analysis offers an alternative framework for examining psychopathology by modeling partial symptom associations and estimating their network-level influence, especially for detecting complex cross-domain relationships that traditional models may overlook [29–33]. Nevertheless, current network-based investigations have largely overlooked clinical adolescent populations, focusing instead on adults or community samples [11, 23]. Research targeting treatment-naïve adolescents in the early phases of depressive illness remains especially limited.

To our knowledge, this is the first study applying symptom network methods to examine somatic and depressive symptoms under different suicide risk indicators in treatment-naïve adolescents with first-episode depression. We employed network analysis alongside validated clinical scales to examine associations within and between symptom domains under different suicide risk indicators, including passive suicidal ideation (PSI), active suicidal ideation (ASI), and suicide attempts (SA).

The principal objective was to identify central and predictable somatic symptom nodes and to describe their structural prominence within the network. We further examined whether distinct patterns of structural heterogeneity in symptom associations were observed under different suicide risk indicators, with a particular focus on somatic symptoms. By modeling somatic symptoms within suicide risk networks in treatment-naïve adolescents, this study provides descriptive evidence relevant to current research on adolescent suicide risk.

## Methods

### Participants

**Fig. 1:**
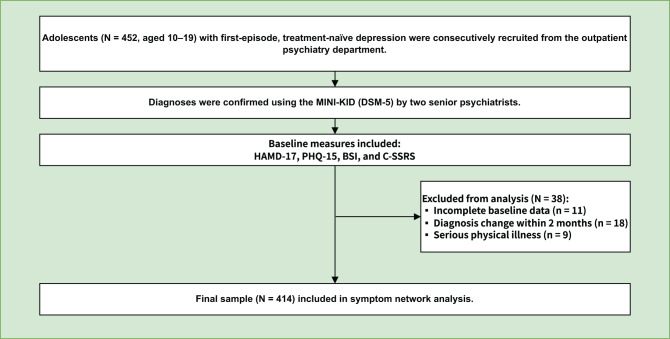
Flow chart of sample inclusion

Adolescents aged 10–19 years were recruited consecutively from the psychiatric outpatient services of the First Affiliated Hospital of Chongqing Medical University between May 2020 and November 2024. All individuals met DSM-5 criteria for a first depressive episode and had no prior exposure to psychotropic medication or psychological treatment. The diagnostic assessments were completed independently by two board-certified psychiatrists, using the Mini-International Neuropsychiatric Interview for Children and Adolescents (MINI-KID) in accordance with DSM-5 diagnostic standards [34]. The MINI-KID was applied uniformly across all participants, including those aged 18–19 years, to ensure diagnostic consistency across the sample. Only individuals currently experiencing a major depressive episode were eligible; those with other depressive disorders, such as persistent depressive disorder, were not included. The recruitment and evaluation process is illustrated in Fig. 1. Participants were excluded if baseline data were missing (*n* = 11), if hypomanic symptoms or other psychiatric disorders were identified within two months of the baseline diagnostic interview (*n* = 18), or if serious physical illness was present (*n* = 9). After exclusion criteria were applied, 414 adolescents with a confirmed diagnosis of depression comprised the final analytic sample. The study protocol was approved by the Human Research and Ethics Committee of the First Affiliated Hospital of Chongqing Medical University (Approval No. 2020–97-2). Written informed consent was obtained from all subjects and their legal guardians in accordance with institutional regulations for the protection of human research subjects.

### Measurements

#### The 17-item Hamilton Rating Scale for depression

Depression severity was assessed using the 17-item Hamilton Rating Scale for Depression (HAMD-17), a clinician-administered instrument widely used in clinical and research settings [35]. Based on Cleary’s factor analytic model, the HAMD-17 can be organized into five distinct dimensions: (1) Anxiety/Somatization (including hypochondriasis, general somatic symptoms, somatic anxiety, psychic anxiety, gastrointestinal symptoms, and insight); (2) Weight loss; (3) Cognitive disturbance (including suicide, guilt, and agitation); (4) Psychomotor retardation (including genital symptoms, retardation, work and interests, and depressed mood);(5) Sleep disturbance (including initial, middle, and delayed insomnia) [36]. Consistent with previous studies, the suicide item (item 3) was excluded from the network to avoid conceptual redundancy with the Beck Scale for Suicide Ideation (BSI) suicide item [37].

#### The Patient Health Questionnaire-15

The Patient Health Questionnaire-15 (PHQ-15) is a validated self-report scale frequently applied in clinical studies for assessing the severity of somatic symptoms over the past four weeks [38]. It evaluates 15 commonly reported physical symptoms. A four-factor structure identified by Witthöft has been empirically validated, comprising: (1) Gastrointestinal (including stomach pain, sexual pain or problems, constipation, and nausea); (2) Fatigue (including tiredness or low energy, and trouble sleeping); (3) Cardiopulmonary (including chest pain, dizziness, palpitations, and shortness of breath); (4) Pain (including back pain, limb pain, and headache) [30, 31]. To prevent measurement redundancy, item 15 (“trouble sleeping”) was excluded to avoid conceptual overlap with the sleep disturbance dimension (D5) of the HAMD-17.

#### The Beck scale for suicide ideation

Suicidal ideation intensity within the past week was measured using the validated Beck Scale for Suicide Ideation (BSI) [39, 40]. Following previous empirical work, two dimensions of suicidal ideation were derived from specific BSI items. Items 1, 2, 3 and 5 were combined to create a passive suicidal ideation (PSI) index, and the sum of scores was between 0 and 8 [41]. Active suicidal ideation (ASI) was calculated by adding Items 4, 12, 13, 15, and 16 and therefore yielded a sum score ranging from 0 to 10. The higher the score on each dimension, the more severe the individual’s suicidal ideation.

#### The Columbia-Suicide Severity Rating Scale (C-SSRS)

Lifetime suicide history was assessed using the Columbia–Suicide Severity Rating Scale (C-SSRS) [42]. For the purposes of network estimation, suicide attempts were coded as a binary variable. Responses were dichotomized as 0 (“No”) or 1 (“Yes”). Fine-grained ordinal classifications of suicidal behavior were not included in the network model, because including many ordered categories in mixed graphical models may complicate interpretation without improving the structure of the model [43].

### Statistical analysis

#### Descriptive statistics

Participant characteristics were summarized using descriptive statistics and are presented as mean ± standard deviation (SD) for continuous variables and frequency percentages for categorical measures. Comparisons were performed between adolescents with and without suicide attempts (SA vs non-SA). Intergroup differences were assessed through parametric (independent samples t-test), nonparametric (Mann–Whitney U test), or contingency-based (chi-square test) approaches, depending on variable distribution normality and measurement scale properties. Statistical significance was defined a priori as a two-tailed α-level < 0.05.

#### Network analysis

A symptom network was constructed to examine the network structure among depressive symptoms, somatic symptoms, and suicide-related indicators in treatment-naïve adolescents experiencing a first depressive episode. The network consisted of 12 nodes representing symptom dimensions and suicide-related indicators. Depressive symptoms were modeled using five HAMD-17 factor scores: Anxiety/Somatization (D1), Weight Loss (D2), Cognitive Disturbance (D3), Psychomotor Retardation (D4), and Sleep Disturbance (D5). Somatic symptom nodes derived from the PHQ-15 included Gastrointestinal symptoms (G), Fatigue (F), Cardiopulmonary symptoms (CP), and Pain (P). Suicidal ideation was represented by BSI-derived passive (PSI) and active (ASI) ideation nodes, and suicide attempts (SA) were included as a binary lifetime indicator. A Mixed Graphical Model (MGM) approach was adopted for network inference, providing a principled solution for capturing conditional dependencies across continuous and discrete binary variable domains [43]. Network topology was visualized using the *qgraph* package (R environment), with edge thickness reflecting the strength of regularized partial correlations between nodes [44, 45]. To enhance sparsity and interpretability, the Graphical LASSO was applied [46, 47]. The best-fit tuning parameter was identified through 10-fold cross-validation, minimizing potential overfitting.

#### Node centrality and predictability

Centrality indices were computed via *qgraph*. To determine strength centrality, we summed the absolute edge weights that link the node to all directly connected nodes. Expected Influence (EI), accounting for both negative and positive weights, served as a refined centrality metric in mixed-edge networks [44]. EI values were used to reflect overall nodal impact across the symptom network. In light of negative edge weights, EI was selected as the principal indicator of centrality in this analysis. Nodes with higher EI values were considered more influential. Alongside strength and expected influence, closeness and betweenness centrality were additionally estimated and are reported for completeness. Given their limited stability in symptom-level psychological networks, these indices were not used for substantive interpretation [44, 48].

Node predictability, computed via *mgm*, reflects the explained variance by directly linked neighbors [49]. For the binary node representing suicide attempts (SA), predictability was assessed via classification accuracy [44, 48]. Predictability was estimated only for continuous nodes in the network [50, 51]. Node predictability was illustrated with pie charts.

#### Stability of edge weights

Edge weight stability was assessed by generating 1,000 nonparametric bootstrap samples with the *mgm* package’s resample function. For each edge, the 5th and 95^th^ percentile intervals and the proportion of non‑zero bootstrap estimates were reported [44]. We used *bootnet* to evaluate the reliability of EI metrics via correlation stability coefficients (CS). CS quantifies network robustness as the upper bound of case exclusion proportions preserving ≥ 0.5 Pearson correlation (ρ) between original and bootstrapped EI centrality values. CS values above 0.25 reflect acceptable stability, while values above 0.50 suggest good stability [44].

## Results

### Sociodemographic and clinical characteristics

The final sample included 414 adolescents (mean age = 15.21 years, SD = 1.78), of whom 78.3% were female. Approximately 8.7% of participants reported a family history of depression, and 71.3% had previously engaged in self-harm. The mean age at depression onset was 13.42 years (SD = 2.68). Participants were grouped by history of suicide attempts, and their demographic and clinical characteristics were contrasted accordingly (Table 1).

**Table 1:** Sample demographics and clinical features by suicide attempt status (*N* = 414)

Characteristic	Attempted suicide			*p*-value
	Overall (*N* = 414^)^	Non- SA(*N* = 293^)^	SA(*N* = 121^)^	
Age(years)	15.21 ± 1.78	15.32 ± 1.79	14.95 ± 1.72	0.047
Sex				0.030
Female	324 (78.3%)	221 (75.4%)	103 (85.1%)	
Male	90 (21.7%)	72 (24.6%)	18 (14.9%)	
Family history				0.854
no	378 (91.3%)	268 (91.5%)	110 (90.9%)	
Yes	36 (8.7%)	25 (8.5%)	11 (9.1%)	
Smoking history				0.323
no	369 (89.1%)	264 (90.1%)	105 (86.8%)	
Yes	45 (10.9%)	29 (9.9%)	16 (13.2%)	
Drinking history				0.903
no	320 (77.3%)	226 (77.1%)	94 (77.7%)	
Yes	94 (22.7%)	67 (22.9%)	27 (22.3%)	
History of self-harm				<0.001
no	119 (28.7%)	101 (34.5%)	18 (14.9%)	
Yes	295 (71.3%)	192 (65.5%)	103 (85.1%)	
Age at onset(years)	13.42 ± 2.68	13.56 ± 2.85	13.09 ± 2.21	0.006

Note. SA = suicide attempt; results are given as mean ± SD or n (%). Group differences tested via t-tests or chi-square tests

Mean values for overall HAMD-17 and PHQ-15 scores were calculated as 21.2 (SD = 5.2) and 16.4 (SD = 5.9), respectively. Mean scores for PSI and ASI were 4.05 (SD = 2.34) and 3.07 (SD = 2.37), respectively. A total of 121 adolescents reported suicide attempts. Table 2 presents key symptom features and their corresponding predictability estimates from the network analysis.

**Table 2:** Symptom node metrics and predictability estimates (*N* = 414)

Characteristic	*N* = 414	Predictability (R^2^)/Classification Accuracy
PSI	4.05 ± 2.34	0.35
ASI	3.07 ± 2.37	0.32
SA	121 (29.2%)	NA^*^
HAMD-17	21.2 ± 5.2	-
D1	1.09 ± 0.39	0.27
D2	0.40 ± 0.67	0.04
D3	1.19 ± 0.60	0.15
D4	1.70 ± 0.41	0.18
D5	0.98 ± 0.58	0.16
PHQ-15	16.4 ± 5.9	-
G	0.87 ± 0.46	0.37
F	1.79 ± 0.43	0.04
CP	1.16 ± 0.54	0.49
P	3.06 ± 1.65	0.47

Note. PSI, passive suicidal ideation; ASI, active suicidal ideation; SA, suicide attempts; D1, Anxiety/Somatization; D2, Weight loss; D3, Cognitive disturbance; D4, Psychomotor retardation; D5, Sleep disturbance; G, Gastrointestinal; F, Fatigue; CP, Cardiopulmonary; P, Pain

*Predictability was not estimated for binary nodes

-HAMD-17 and PHQ-15 totals not included in the network. Values shown as mean ± SD if not otherwise noted

### Network structure estimation and Visualization

#### Symptom network and graphical output

**Fig. 2:**
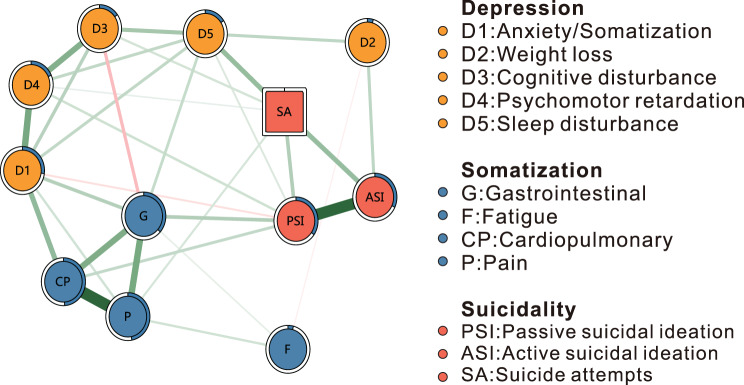
The estimated symptom network (*n* = 414). Edge color denotes association direction (green = positive, red = negative); edge thickness corresponds to association strength. Node predictability is visualized as pie charts, with values reported in Table 2. All edge weights are presented in supplementary Table S1. PSI, passive suicidal ideation; ASI, active suicidal ideation; SA, suicide attempts; D1, Anxiety/Somatization; D2, weight loss; D3, Cognitive disturbance; D4, Psychomotor retardation; D5, Sleep disturbance; G.Gastrointestinal; F, Fatigue; CP, Cardiopulmonary; P, pain

The network model included 12 symptoms spanning depressive, somatic, and suicide risk domains. As shown in Fig. 2, the network was estimated in treatment-naïve adolescents with depression. Of the 66 possible edges, 31 (47.0%) showed non-zero partial correlations, reflecting conditional associations among symptoms. Two negative edges were identified: between Weight loss (D2) and Fatigue (F), and between Cognitive disturbance (D3) and Gastrointestinal (G). All other edges were positive. Cardiopulmonary symptoms were specifically linked to passive suicidal ideation, whereas pain symptoms were selectively associated with suicide attempts, and showed no connections to other suicide-related symptoms.

#### Centrality and predictability

**Fig. 3:**
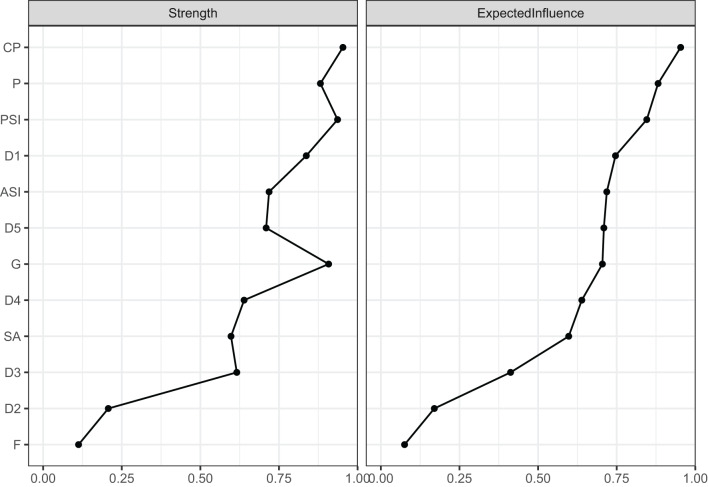
Centrality indices for each node in the estimated symptom network. Note:Both strength and expected influence (EI) are presented. Because negative edge weights (<0) were considered, EI was used as the primary centrality index. Higher values indicate greater importance of the node within the network. Nodes are arranged in descending order of expected influence. PSI, passive suicidal ideation; ASI, active suicidal ideation; SA, suicide attempts; D1, Anxiety/Somatization; D2, weight loss; D3, Cognitive disturbance; D4, Psychomotor retardation; D5, Sleep disturbance; G.Gastrointestinal; F, Fatigue; CP, Cardiopulmonary; P, pain

Centrality analysis revealed that cardiopulmonary (CP) exhibited the highest expected influence (CP; EI = 0.95), followed by Pain (P; EI = 0.88), passive suicidal ideation (PSI; EI = 0.85), and Anxiety/Somatization (D1; EI = 0.75). In contrast, Weight loss (D2) showed the lowest EI (EI = 0.17), suggesting limited network involvement (Fig. 3). Supplementary Figure S1 illustrates the centrality stability coefficient (CS) for expected influence, which was 0.44, indicating acceptable stability and moderate reliability of the centrality estimates.

As shown in Table 2, the cardiopulmonary (CP) and pain (P) nodes exhibited the highest predictability, with values of 49.1% and 47.1%, respectively. Gastrointestinal (G; 36.5%) and passive suicidal ideation (PSI; 34.7%) demonstrated moderate predictability. Notably, the suicide attempts (SA) node demonstrated zero predictability. Closeness and betweenness centrality estimates are provided in Supplementary Figure S2. Predictability was not estimated for the suicide attempt (SA) node, as it was modeled as a binary variable.

#### Edge weight precision and stability

The consistency and accuracy of edge weight estimates were examined. Supplementary Figure S3 presents the 95% bootstrapped percentile intervals across network edges, displaying the distribution of resampled values, the 5th and 95th percentiles, and the proportion of iterations in which each connection remained non-zero. Because regularization techniques shrink edge estimates toward zero, confidence intervals that include zero do not necessarily imply null associations [44, 52].

## Discussion

To our knowledge, this is among the first studies to apply symptom network analysis to depressive, somatic, and suicide risk indicators in treatment-naïve adolescents with first-episode depression. Cardiopulmonary (CP) and pain (P) emerged as central nodes within the symptom network. CP exhibited a weak association with PSI, while P showed a weak association with SA. These patterns illustrate how somatic symptom dimensions are structurally positioned within the broader configuration of suicide risk indicators, within a cross-sectional symptom network, without addressing developmental ordering or causal processes. Given the cross-sectional design and the Asian clinical context, longitudinal and cross-cultural research are needed to clarify symptom dynamics over time.

### Cardiopulmonary and pain symptoms as central nodes

Within the estimated network, CP and P were positioned among the more central nodes in relation to suicide-related indicators. A weak positive association was observed between CP and PSI, indicating a closer association with ideational rather than behavioral suicide risk indicators [53]. Pain symptoms, in contrast, exhibited weak associations with suicide attempts (SA), a pattern that has also been reported in longitudinal cohort studies of adolescents [22, 26, 54] Pain-related processes have been discussed in theoretical accounts such as the Interpersonal Theory of Suicide, particularly in relation to mechanisms proposed to differentiate suicidal ideation to suicidal behavior [55, 56]. However, the present cross-sectional network does not permit direct evaluation of such mechanisms and should be interpreted as a descriptive symptom configuration rather than a test of theoretical models. Accordingly, the observed configuration of CP and P represents descriptive symptom associations within a Chinese adolescent clinical sample, with implications for generalizability to be assessed through future cross-cultural research [57, 58].

### Peripheral position of suicide attempts in the symptom network

In our network, the suicide attempts (SA) node was peripheral, showed weak connections to PSI and ASI. In this analysis, SA was defined as a binary indicator reflecting any lifetime attempt, and node predictability was therefore not estimated for this variable. Its peripheral position may be partly attributable to this operationalization, as well as to the relatively small proportion of participants reporting prior attempts. In addition, contextual and behavioral influences relevant to suicidal behavior were not explicitly represented among the network nodes [59, 60]. Overall, the present network depicts a cross-sectional configuration in which associations among suicide risk–related indicators are generally weak and structurally limited. Longitudinal designs may be better suited to examining how symptom interactions evolve over time and how behavioral or psychosocial factors contribute to individual risk trajectories [61].

### Limitations

This study has several noteworthy limitations. First, because of the cross-sectional design, the present findings cannot address temporal ordering or causal relationships among symptoms. Second, depressive symptoms were modeled at the factor level according to the HAMD-17 structure, a strategy that improved network stability but reduced symptom-level granularity and precluded analysis of some core affective features or severity indicators as standalone nodes. Accordingly, the present findings are best interpreted as describing associative patterns among symptom dimensions, rather than diagnostic classifications or severity-based differentiation. Third, suicide attempts were operationalized as a lifetime binary indicator, whereas suicidal ideation was assessed over the past week—a temporal mismatch that may have weakened observed associations involving SA. Finally, the lack of broader suicide-relevant constructs (e.g., early adversity, impulsivity, emotional dysregulation), combined with the moderate sample size, may constrain interpretability, generalizability, and the feasibility of subgroup analyses [7]. Accordingly, the absence of these developmental and regulatory factors represents an important boundary of the present network and should be considered when interpreting the findings.

## Conclusions

In this cross-sectional symptom network of treatment-naïve adolescents with first-episode depression, cardiopulmonary and pain symptoms occupied structurally prominent positions and showed different patterns of association with suicide risk indicators. Taken together, these findings suggest symptom networks can serve as a descriptive tool for conceptually organizing somatic symptoms within heterogeneous suicide risk profiles in adolescent depression. Rather than advancing causal interpretations, the present network serves as a descriptive organizational framework that can inform future longitudinal research on the temporal dynamics and clinical relevance of suicide-related symptom patterns in adolescents.

## Electronic supplementary material

Below is the link to the electronic supplementary material.

Supplementary material 1

## Data Availability

The anonymized dataset supporting the findings of this study is available from the corresponding author upon reasonable request.

## Abbreviations

PSI: Passive suicidal ideation
ASI: Active suicidal ideation
SA: Suicide attempts
CP: Cardiopulmonary
P: Pain
G: Gastrointestinal
F: Fatigue
D1: Anxiety/Somatization
D2: Weight loss
D3: Cognitive disturbance
D4: Psychomotor retardation
D5: Sleep disturbance
