# Supplementary material for: Symptom network analysis of depressive and somatic symptoms and suicide risk indicators in treatment-naïve adolescents with first-episode depression
Source: BMC Psychiatry. 2026 Apr 21;26:447. doi: 10.1186/s12888-026-08074-1 (PMC13238123; doi:10.1186/s12888-026-08074-1)
Supplement: Supplementary file 1 — Supplementary material 1 [file 12888_2026_8074_MOESM1_ESM.docx]

**TableS1**

**Estimated edge weights (n=414)**

|  | SA | PSI | ASI | D1 | D2 | D3 | D4 | D5 | G | F | CP | P |
| --- | --- | --- | --- | --- | --- | --- | --- | --- | --- | --- | --- | --- |
| SA |  |  |  |  |  |  |  |  |  |  |  |  |
| PSI | 0.12 |  |  |  |  |  |  |  |  |  |  |  |
| ASI | 0.17 | 0.46 |  |  |  |  |  |  |  |  |  |  |
| D1 | 0.00 | 0.05 | 0.00 |  |  |  |  |  |  |  |  |  |
| D2 | 0.00 | 0.00 | 0.09 | 0.00 |  |  |  |  |  |  |  |  |
| D3 | 0.06 | 0.00 | 0.00 | 0.10 | 0.00 |  |  |  |  |  |  |  |
| D4 | 0.03 | 0.07 | 0.00 | 0.24 | 0.00 | 0.21 |  |  |  |  |  |  |
| D5 | 0.17 | 0.04 | 0.00 | 0.09 | 0.09 | 0.14 | 0.09 |  |  |  |  |  |
| G | 0.00 | 0.12 | 0.00 | 0.11 | 0.00 | -0.10 | 0.00 | 0.08 |  |  |  |  |
| F | 0.00 | 0.00 | 0.00 | 0.00 | -0.02 | 0.00 | 0.00 | 0.00 | 0.03 |  |  |  |
| CP | 0.00 | 0.09 | 0.00 | 0.18 | 0.00 | 0.00 | 0.00 | 0.00 | 0.22 | 0.00 |  |  |
| P | 0.05 | 0.00 | 0.00 | 0.06 | 0.00 | 0.00 | 0.00 | 0.00 | 0.24 | 0.06 | 0.46 |  |

Note:PSI, passive suicidal ideation; ASI, active suicidal ideation; SA, suicide attempts; D1, Anxiety/somatization; D2, weight loss; D3, Cognitive disturbance; D4, Psychomotor retardation; D5, Sleep disturbance; G.Gastrointestinal; F, Fatigue; CP, Cardiopulmonary; P, Pain.

**
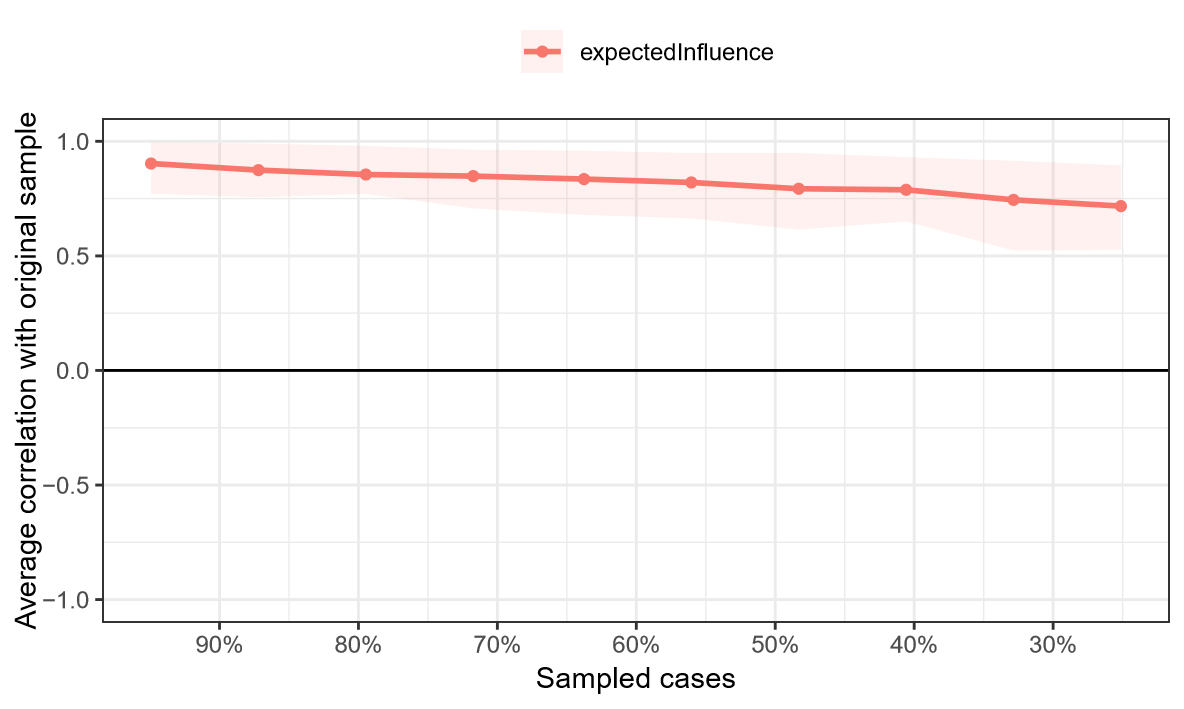
**

**Figure S1.** Centrality stability (CS) coefficient for expected influence (EI) in the symptom network. Stability was assessed using 1,000 nonparametric bootstrap samples with the bootnet package. The CS coefficient reflects the maximum proportion of cases that can be dropped while maintaining a Pearson correlation of at least 0.5 between the original and subset EI estimates. A CS value of 0.44 indicates acceptable stability and moderate reliability of the expected influence centrality metrics, supporting the robustness of the key symptom nodes identified in this network.

**
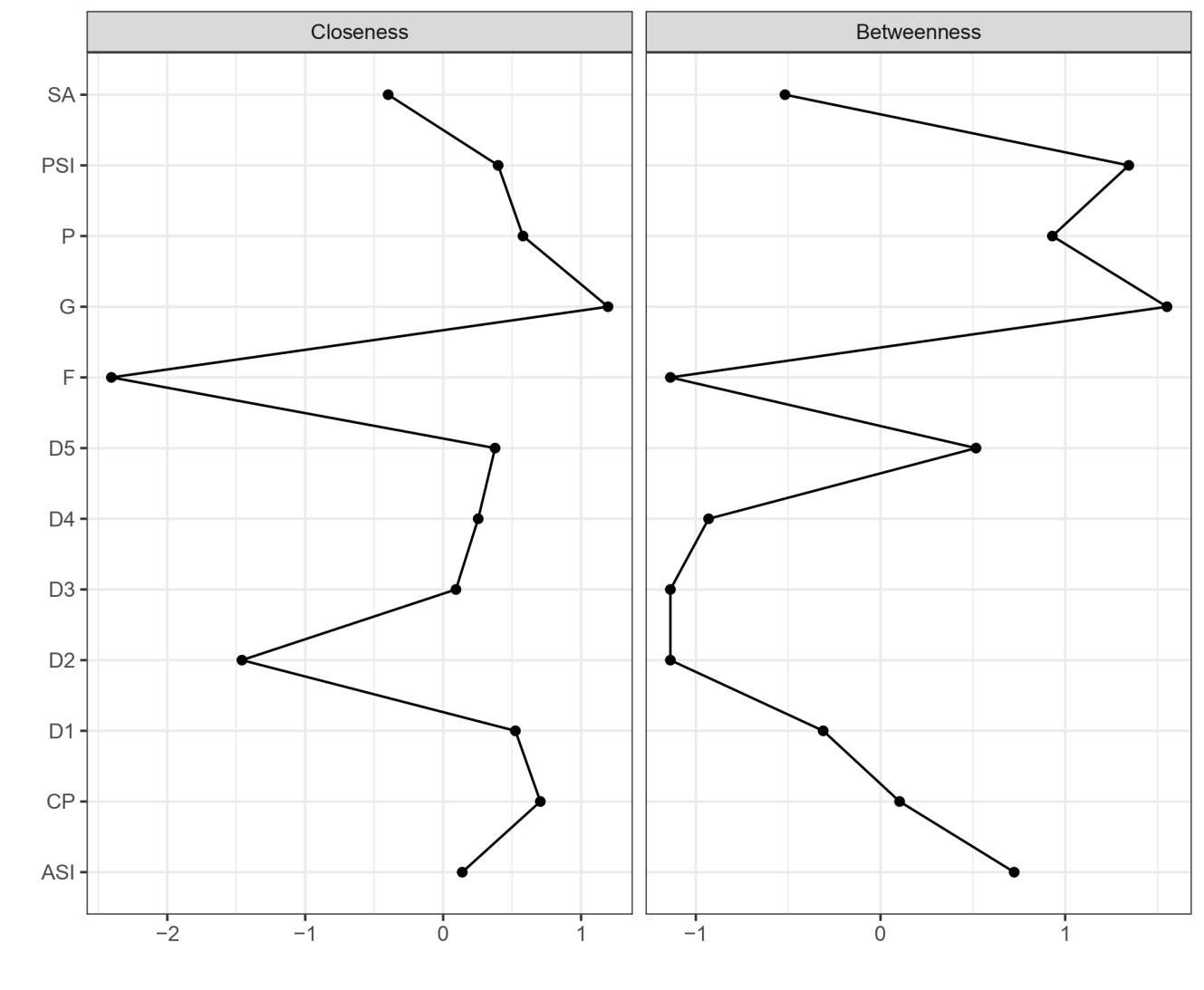
**

**Figure S2**. **Closeness and Betweenness Centrality for the Symptom Network**.

*Closeness centrality* reflects a node’s average shortest-path distance to all other nodes, serving as a graph-theoretical indicator of relative proximity within the estimated network. *Betweenness centrality* reflects how frequently a node lies on the shortest paths between other node pairs, describing its positional role in the network topology.

**Note:** PSI, passive suicidal ideation; ASI, active suicidal ideation; SA, suicide attempts; D1, anxiety/somatization; D2, weight loss; D3, cognitive disturbance; D4, psychomotor retardation; D5, sleep disturbance; G, gastrointestinal; F, fatigue; CP, cardiopulmonary; P, pain.


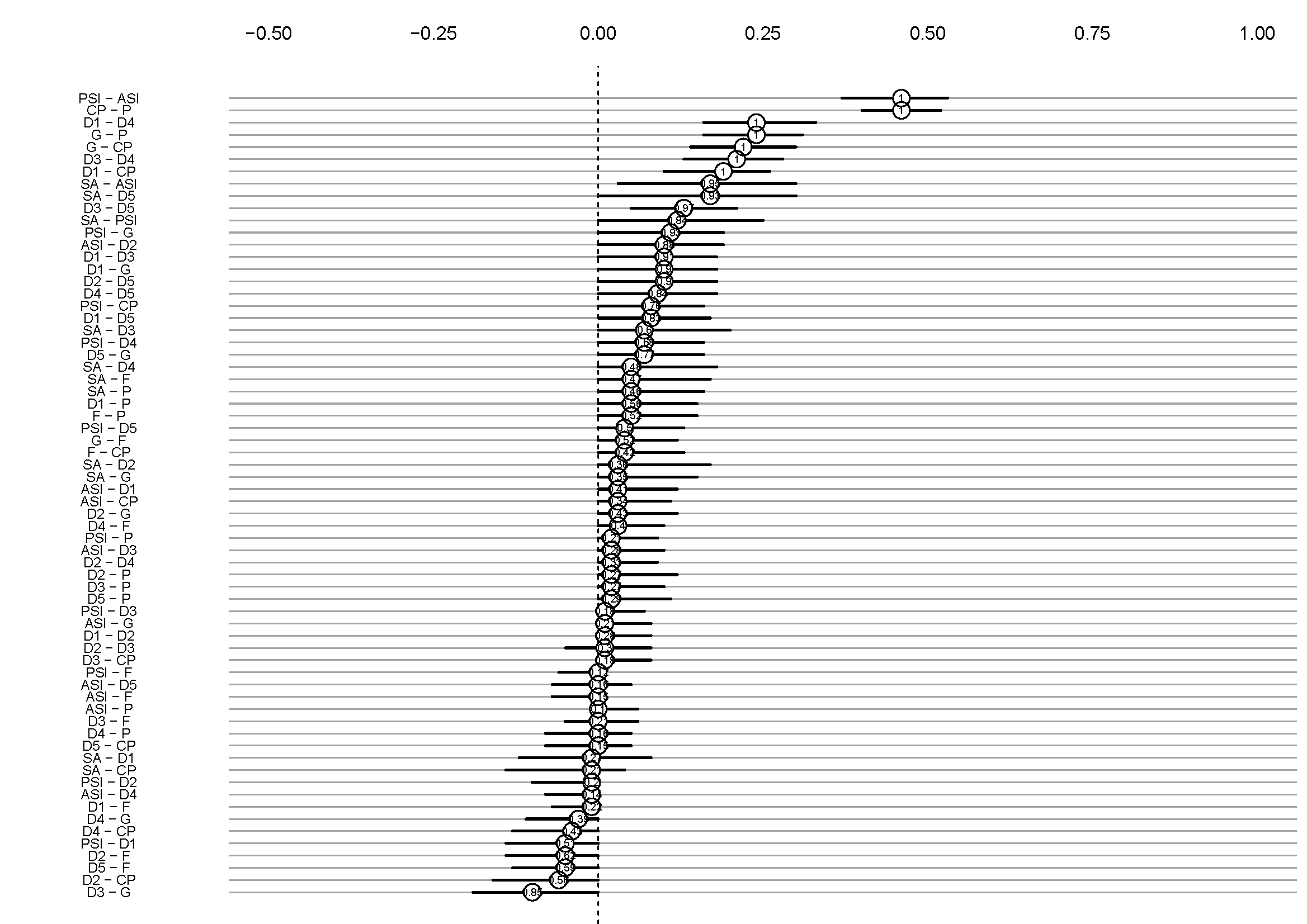


**Figure S3.** Stability of edge weight estimates in the symptom network, based on 1,000 bootstrap resamples. The numbers within each edge represent the proportion of bootstrap samples where the edge was retained as nonzero, reflecting the robustness of the estimated connections.
